# Reporter lines based on the *gexp02* promoter enable early quantification of sexual conversion rates in the malaria parasite *Plasmodium falciparum*

**DOI:** 10.1038/s41598-019-50768-y

**Published:** 2019-10-10

**Authors:** Harvie P. Portugaliza, Oriol Llorà-Batlle, Anna Rosanas-Urgell, Alfred Cortés

**Affiliations:** 1 ISGlobal, Hospital Clinic – Universitat de Barcelona, Barcelona, 08036 Catalonia Spain; 2 Department of Biomedical Sciences, Institute of Tropical Medicine, Antwerp, 2000 Belgium; 3 Department of Global Health, Amsterdam University Medical Centers, location Academic Medical Center, University of Amsterdam, Amsterdam, 1105 AZ The Netherlands; 4 ICREA, Barcelona, 08010 Catalonia Spain

**Keywords:** Malaria, Parasite biology, CRISPR-Cas9 genome editing

## Abstract

Transmission of malaria parasites from humans to mosquito vectors requires that some asexual parasites differentiate into sexual forms termed gametocytes. The balance between proliferation in the same host and conversion into transmission forms can be altered by the conditions of the environment. The ability to accurately measure the rate of sexual conversion under different conditions is essential for research addressing the mechanisms underlying sexual conversion, and to assess the impact of environmental factors. Here we describe new *Plasmodium falciparum* transgenic lines with genome-integrated constructs in which a fluorescent reporter is expressed under the control of the promoter of the *gexp02* gene. Using these parasite lines, we developed a sexual conversion assay that shortens considerably the time needed for an accurate determination of sexual conversion rates, and dispenses the need to add chemicals to inhibit parasite replication. Furthermore, we demonstrate that *gexp02* is expressed specifically in sexual parasites, with expression starting as early as the sexual ring stage, which makes it a candidate marker for circulating sexual rings in epidemiological studies.

## Introduction

Malaria disease is produced by repeated cycles of asexual parasite replication in the human blood. During the ~48 h intraerythrocytic asexual replication cycle, parasites develop through the ring, trophozoite and schizont stages. This is followed by infected erythrocyte rupture and egress of merozoites, which invade erythrocytes to start a new round of multiplication.

The spreading of malaria between humans is mediated by *Anopheles* mosquitoes. Human to vector transmission requires that a subpopulation of the parasites abandons the asexual cycle and differentiates into non-replicative male or female sexual forms termed gametocytes. In the case of *P*. *falciparum*, which is responsible for the more severe forms of human malaria, parasites that undergo sexual development mature through the morphologically distinct gametocyte stages I to V over ~10 days. Only mature stage V male and female gametocytes are infectious to mosquitoes, where they are activated to form gametes^1,2^. Given their essential role for malaria transmission, gametocytes are considered a priority target for strategies aimed at eliminating malaria.

The initial steps of *P*. *falciparum* sexual differentiation are regulated by PfAP2-G, a transcription factor of the ApiAP2 family that drives the expression of early gametocyte genes^3–7^. In asexual parasites, the gene encoding this transcription factor, *pfap2-g*, adopts a heterochromatic conformation that results in epigenetic silencing, whereas activation to initiate sexual development requires a transition to a euchromatic state^3,5,8,9^. This transition is facilitated by the gametocyte development 1 (PfGDV1) protein, a recently identified upstream regulator of *pfap2-g*^10^. Expression of PfAP2-G marks the sexually committed stage, which according to recently proposed definitions^11^ is a cell state that deterministically results in sexual development at a later point. Commitment is followed by sexual conversion, marked by the expression of gametocyte-specific proteins absent from any replicating blood stages. While previously it was thought that an additional round of replication after commitment was an obligate step^12^, recent research in *P*. *falciparum* and the murine malaria parasite *P*. *berghei* demonstrated that sexual conversion can also occur within the same cycle of commitment^11,13^. For both sexual conversion pathways, termed same cycle conversion (SCC) and next cycle conversion (NCC)^11^, conversion results in the formation of sexual ring stages that then develop into stage I to V gametocytes. The only sexual stages present in the circulation are sexual rings and mature stage V gametocytes^14,15^, whereas stage I-IV gametocytes are sequestered in tissues such as the bone marrow^1,6,16,17^.

While sexual stages mediate transmission, the asexual cycle results in within-host parasite expansion, providing the opportunity to generate more sexual forms. The relative investment in multiplication and sexual differentiation is tightly adjusted to ensure long-term survival, and in the case of *P*. *falciparum* only a small fraction of the parasites (typically <10%) differentiate into sexual forms at each cycle of multiplication^18,19^. The sexual conversion rate, defined as the proportion of parasites that become gametocytes at each replication cycle, underlies the trade-off between growth in the same host and transmission. Non-induced (baseline) sexual conversion rates vary between different parasite lines^3^, and conversion can be induced by external cues. Several conditions, including drug treatment, have been proposed to stimulate sexual conversion^2,5,6,20^, but depletion of the serum component lysophosphatidylcholine (LysoPC) stands out as a highly reproducible induction method that is likely relevant during human infection^21^. Addition or removal of choline, involved in the same metabolic pathway as LysoPC, can be used as a convenient alternative to repress or induce sexual conversion under culture conditions^10,21^.

A common approach to measure sexual conversion rates consists of determining the gametocytemia of a culture relative to the initial rings parasitemia of the synchronized culture from which the gametocytes originated^3,11,14,22^. This reflects the proportion of sexual vs total rings in the initial culture. Gametocytemia is typically measured by light microscopy analysis of Giemsa-stained smears, which is laborious and has limited accuracy because gametocytemia is typically much lower than the asexual parasitemia. An additional limitation of this assay is that gametocytemia is typically measured >3 days after seeding the assay, as unambiguous morphological identification is not possible until gametocytes reach stage II^5,23^. Since the asexual parasites present in the culture continue multiplying every 48 h, to prevent culture collapse and to identify gametocytes more easily, cultures are usually treated with chemicals such as N-acetyl-D-glucosamine (GlcNAc) or heparin^24,25^ that do not kill non-replicating gametocytes but inhibit asexual parasite multiplication. Altogether, this standard assay to determine sexual conversion rates is time-consuming, has limited accuracy, and is not suitable for high-throughput approaches. As an alternative, sexual conversion rates have been measured using immunofluorescence assay (IFA) analysis with antibodies against early gametocyte markers such as Pfs16, but this method still required quantification of the proportion of sexual parasites by fluorescence microscopy^10,26,27^.

Assays that use flow cytometry to quantify gametocytes at an early stage of sexual development are ideally suited to accurately determine sexual conversion rates. Transgenic parasite lines expressing fluorescent proteins under the control of promoters from genes expressed in early gametocytes such as *pfg14-744*, *pfg14-748*, *etramp10*.*3*, *pfg27* and *pfs16* have been described^28–35^. However, in all cases the reporter constructs were maintained episomally, implying that continuous drug pressure was required to maintain the episome. Even in the presence of selective pressure, some parasites lose the episome at each division^30,35^, and some drugs may affect sexual conversion, resulting in confounding effects^2,5,6,20^. Furthermore, the promoters used do not have high activity until stage I or II of gametocyte development, and are inactive or expressed at low levels at the sexual ring stage.

Beyond the early gametocyte markers that have been known for years such as Pfs16, Pfg27 or Pfg14.744^34,36,37^, several new early gametocyte markers have been recently identified using genome-wide approaches^3,4,7,10,21,26,32,38^. New gametocyte markers with an earlier onset of expression during sexual development, already present in sexual rings, would be valuable not only to study sexual conversion *in vitro*, but also for epidemiological studies. Currently, the only well-validated specific transcript marker for sexually committed stages and sexual rings is *pfap2-g*. The expression of the protein GEXP05 starts as early as the sexual ring stage^39,40^, but the high rate of *gexp05* expression in asexual parasites^11,40^ suggests that assays based on these transcripts may not be sufficiently specific for sexual parasites. Among the new early gametocyte markers that have not been characterized in detail, *gexp02*^32^ was consistently identified as one of the earliest responders to PfAP2-G activation^10,11,15,26,41^. Additionally, using a transgenic parasite line with inducible *pfap2-g* expression we also found that *gexp02* is one of the very few genes that is expressed at high levels in response to PfAP2-G activation as early as the sexual ring stage (Llorà-Batlle *et al*., manuscript in preparation).

To develop a robust and simple sexual conversion assay, here we generated stable *P*. *falciparum* lines with a genome-integrated tandem Tomato (tdTom) fluorescent marker gene controlled by early gametocyte promoters. We show that transgenic lines using the *gexp02* promoter enable specific identification of sexual stages by flow cytometry or immunofluorescence assay (IFA) as early as the sexual ring stage, thereby shortening considerably the duration of the sexual conversion assay. We also show that *gexp02* transcripts provide a new marker to specifically detect sexual parasites as early as the sexual ring stage.

## Results and Discussion

### Generation of early gametocyte-reporter transgenic lines

The reporter lines were generated from two high gametocyte producer *P*. *falciparum* culture-adapted lines, NF54 and E5. The NF54 line shows high sexual conversion rates and is commonly used to infect mosquitoes, as it retains the ability to produce infective male and female gametocytes^42^. The E5 line is a subclone of 3D7 (itself a clone of NF54^43^) that has been used to study the early steps of sexual differentiation^3,4,11^. Cultures were maintained in 0.5% Albumax II supplemented with 2 mM choline for non-inducing conditions, and choline was removed to induce conversion^10,21^.

**Figure 1:**
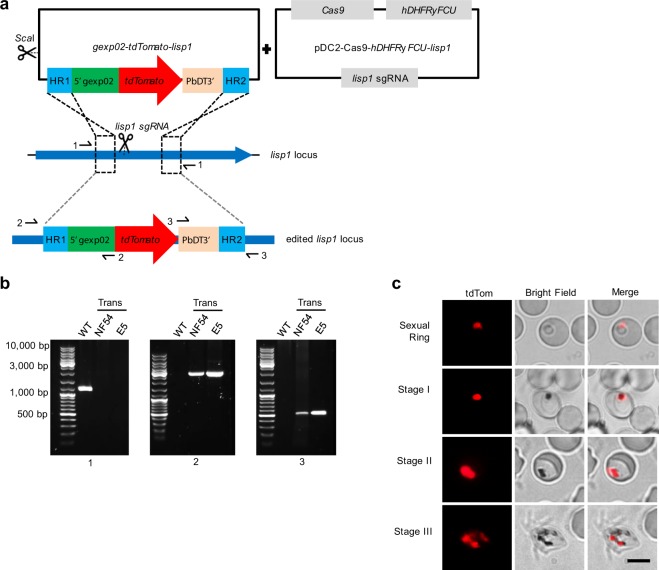
Generation and validation of gametocyte-reporter lines based on the *gexp02* promoter. (**a)** Schematic of the strategy to generate the transgenic lines using the CRISPR/Cas9 system. Half-arrows indicate the position of primers used for diagnostic PCR. **(b)** Diagnostic PCR confirmation of the integration of the *gexp02-tdTomato-lisp1* plasmid at the *lisp1* locus. Numbers at the bottom indicate the primer pair used for each PCR reaction. Genomic DNA from the wild type E5 line (WT) or the NF54 and E5 transgenic lines (Trans) was used. The size of the bands was as expected for the wild-type (1,184 bp for PCR 1) or correctly-edited locus (2,875 bp for PCR 2 and 550 bp for PCR 3). The size of the most intense and top bands of the size marker is indicated. A PCR product was not amplified with primer pair 1 in the transgenic lines, likely because of the too large size of the expected amplification product. **(c)** Live cell fluorescence microscopy analysis of induced (choline-depleted) *NF54-gexp02-Tom* cultures, revealing a subset of tdTom-positive cells consistent with the sexual ring stage (no detectable hemozoin pigment) (top panel). At days 1 to 3 after N-acetyl-D-Glucosamine (GlcNAc) treatment, tdTom signal was observed in parasites morphologically resembling gametocytes at different stages of development (other panels). Scale bar: 5 µm.

Using CRISPR-Cas9 technology, we generated reporter lines with the *gexp02* (PF3D7_1102500) promoter controlling the expression of the fluorescent reporter *tdTomato* (tdTom) integrated in the *lisp1* (PF3D7_1418100) locus (*NF54-gexp02-Tom* and *E5-gexp02-Tom* lines) (Fig. 1a). The *lisp1* gene plays a role in liver stages and is not expressed in blood stage parasites^44^. Diagnostic PCR confirmed correct integration of the construct in both NF54 and E5. In all cases, bands corresponding to the wild type *lisp1* locus were not detected in the transfected parasites, indicating that editing occurred in virtually all parasites (Fig. 1b). Preliminary characterization by live cell fluorescence microscopy revealed that only a subset of the parasites expressed tdTom, consistent with the expected gametocyte-specific expression. A strong signal was observed for parasites that, based on morphology and time of collection, appeared to be at the sexual ring stage or stages I-III of gametocyte development (Fig. 1c and Supplementary Fig. 1a).

We also generated analogous transgenic lines in which the fluorescent marker was under the control of the *etramp10*.*3* (PF3D7_1016900) promoter, which was previously shown to drive strong expression of reporter genes in sexual parasites from stage I/II onwards^28,29,33^. While in these previous studies the plasmids were maintained episomally, we integrated the *etramp10*.*3*-tdTom expression cassette in the NF54 and E5 genomes to generate the *NF54-10*.*3-Tom* and *E5-10*.*3-Tom* lines (Supplementary Fig. 1b). We also generated a reporter line in which tdTom was under the control of the promoter of the early gametocyte marker Pfs16 (*E5-pfs16-Tom*), but preliminary analysis of this line revealed a general fluorescent signal in many multinucleated parasites (i.e. schizonts) that did not demarcate a distinct subset of cells (Supplementary Fig. 1c), so this parasite line was not further characterized. Reporter lines with an episomal *pfs16* promoter driving the expression of a fluorescent marker had been previously reported^30,31^. In one of the studies, fluorescence was observed in a subset of schizonts, which were hypothesized to be sexually committed^31^. However, using specific antibodies the Pfs16 protein was found to be an absolutely specific gametocyte marker expressed from stage I of gametocyte development or even earlier, but not in sexually committed schizonts^11,36^. Altogether, these observations suggest that the *pfs16* promoter may not be fully specific for sexual stages; instead, the absolutely sexual parasite-specific expression of the Pfs16 protein may be conferred by post-transcriptional mechanisms.

### Sexual stage expression of the g*exp02* promoter

**Figure 2:**
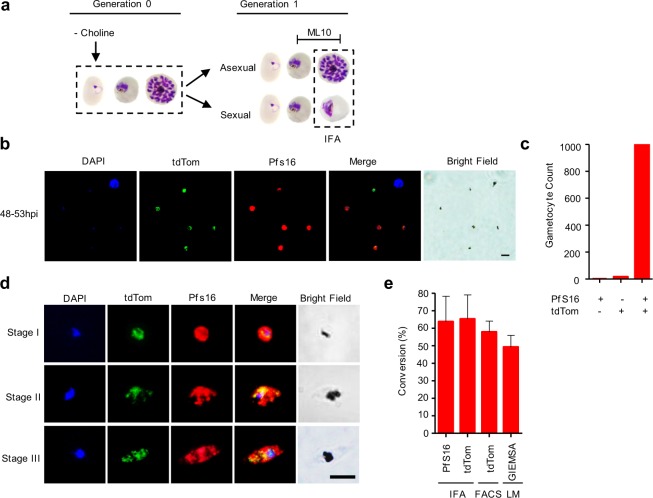
Expression of *gexp02-Tom* in sexual stage parasites. (**a)** Schematic overview of the experiment design with tightly synchronized *NF54-gexp02-Tom* cultures. Sexual conversion was induced by choline-depletion at the ring stage. ML10 was added at ~30–35 h post-invasion (hpi) of the next cycle to prevent schizont rupture. **(b)** Representative immunofluorescence assay (IFA) image of *NF54-gexp02-Tom* cultures at ~48–53 hpi. Mononucleated parasites (stage I gametocytes) express tdTom and Pfs16. Scale bar: 5 µm. **(c)** Distribution of parasites positive for the different markers by IFA (1,176 single-nucleated parasites scored). **(d)** Representative IFA images of gametocytes (stages I-III) showing expression of tdTom and Pfs16 in the same parasites. Scale bar: 5 µm. **(e)** Sexual conversion rates of the *NF54-gexp02-Tom* line as determined by IFA, flow cytometry (FACS) (gametocytemia determined at ~48–53 hpi), and Giemsa-stained blood smears (gametocytemia determined on day 4). Data are presented as the average and s.e.m. of three independent biological replicates.

To confirm the ability of the *gexp02* promoter to identify sexual stages, we analyzed the *NF54-gexp02-Tom* line by IFA, co-staining parasites with antibodies against tdTom and Pfs16. To obtain high levels of gametocytes, we induced sexual conversion by removing choline^10,21^ at the ring stage. At the following cycle, the cGMP-dependent protein kinase (PfPKG) inhibitor ML10 was added to the cultures ~30–35 h post-invasion (hpi) to prevent schizont rupture and reinvasion^45^. IFA analysis was performed when sexual parasites were at stage I of gametocyte development and replicating parasites were mainly at the schizont stage (~48–53 hpi) (Fig. 2a). Essentially all mononucleated parasites expressing endogenous Pfs16 (gametocytes) also expressed tdTom, and vice versa (Fig. 2b,c). However, a distinct small subset of schizonts that may correspond to sexually committed schizonts also expressed tdTom, but not Pfs16 (discussed below in following sections). After treating cultures with GlcNAc, we observed the two proteins co-expressed in developing gametocytes (Fig. 2d). Cross-reactivity of the secondary antibodies was excluded by experiments in which one of the primary antibodies was not added (Supplementary Fig. 2a). As expected from these results, the conversion rate determined by IFA with antibodies against Pfs16 or TdTom was almost identical, and roughly coincided with determinations based on flow cytometry analysis of tdTom or by microscopy analysis of Giemsa-stained smears (Fig. 2e). Altogether, these experiments demonstrate the specificity of our system based on the *gexp02* promoter to quantify gametocytemia. Analysis of sexual conversion rates in the *NF54-10*.*3-Tom* line also revealed consistent results between experiments based on IFA, flow cytometry or Giemsa-stained smears (Supplementary Fig. 2b).

### Flow cytometry-based time-course analysis of *gexp*02-tdTom expression

**Figure 3:**
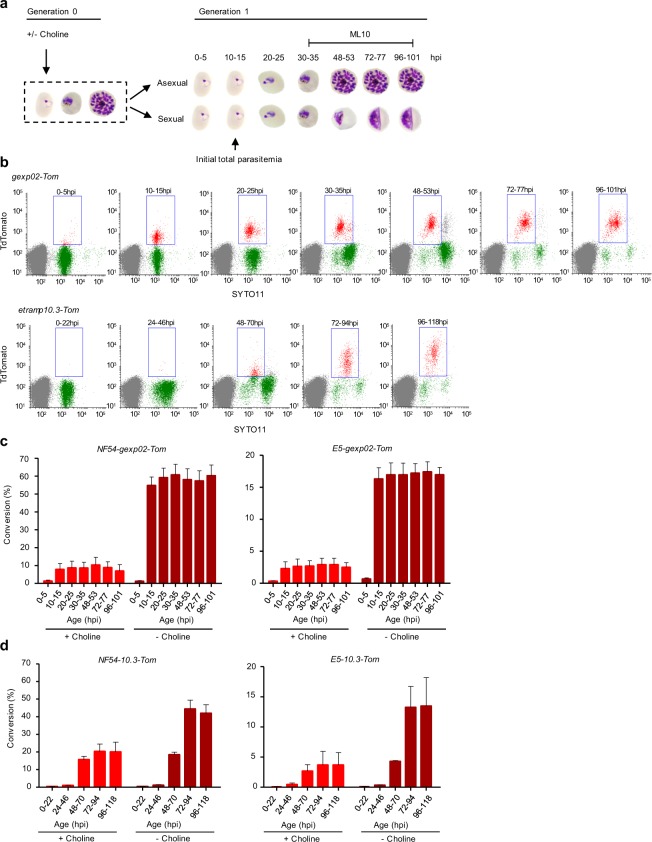
Flow cytometry-based time-course analysis of *gexp02*-tdTom and *etramp10*.*3*-tdTom expression during sexual development. (**a**) Schematic overview of the experiment design. Cultures were either maintained in the presence of choline, or with choline removed at the ring stage to induce sexual conversion. After reinvasion, the proportion of tdTom-positive parasites was determined by flow cytometry at several times (the times shown correspond to experiments with the *gexp02-Tom* lines, which were synchronized to a 5 h age window). ML10 was added at the trophozoite stage (generation 1, as indicated) to prevent schizont rupture and reinvasion. **(b)** Representative flow cytometry SYTO11 (stains DNA) – tdTomato plots for the *NF54-gexp02-Tom* and *NF54-etramp10*.*3-Tom* transgenic lines (- choline condition) at different times after invasion. Gates (rectangles) indicate SYTO11 and tdTomato double-positive parasites. **(c)** Quantification of sexual conversion rates at different times in the *NF54-gexp02-Tom* and *E5-gexp02-Tom* lines, based on detection of tdTomato fluorescence. Conversion rates (%) were determined as the gametocytemia relative to the initial rings parasitemia. Data are presented as the average and s.e.m. of three independent biological replicates. **(d)** Same as panel c, but for the *NF54-etramp10*.*3-Tom* and *E5-etramp10*.*3*-*Tom* lines. Data are presented as the average and s.e.m. of two independent biological replicates.

To identify the earliest time for accurate gametocytemia measurements, we determined the temporal expression dynamics of tdTom driven by the *gexp02* or the *etramp10*.*3* promoters using flow cytometry. After defining the gates of tdTom-specific signal (Supplementary Fig. 3), cultures of the four transgenic lines were Percoll/sorbitol synchronized to a defined 5 h age window (lines based on the *gexp02* promoter) or sorbitol synchronized (lines based on the *etramp10*.*3* promoter) and grown under inducing (- choline) and non-inducing (+choline) conditions^10,21^ (Fig. 3a). In both the *NF54-gexp02-Tom* and *E5-gexp02-Tom* lines, a population of tdTom-positive parasites was evident as early as 10–15 hpi (generation 1), both under inducing and non-inducing conditions, with a relatively stable level of expression until 96–101 hpi (Fig. 3b,c). In contrast, in the *NF54-10*.*3-Tom* and *E5-10*.*3-Tom* lines full expression of the marker was not observed until 72–94 hpi (stage II gametocytes), although weaker signal was already observed in many parasites at 48–70 hpi (Fig. 3b,d). Altogether, these results demonstrate that using the *gexp02* promoter lines, gametocytemia and sexual conversion rates can be accurately measured as early as 10–15 hpi. Since reinvasion does not occur until much later, the use of the *gexp02* promoter dispenses the need to add chemicals to inhibit the growth of asexual parasites.

Of note, the new transgenic lines showed similar sexual conversion rates to their parental lines and were inducible by choline depletion (6 to 7-fold higher conversion under inducing conditions in *gexp02* promoter-based lines) (Fig. 3), demonstrating that their sexual conversion phenotypes were not substantially altered during genome editing. Unexpectedly, the NF54 line used in this study shows an extremely high sexual conversion rate upon induction (~60%), comparable to the conversion rates observed upon depletion of HP1 or the *gdv1* antisense RNA^10,26^. We also observed that the multiplication rate was reduced in choline-depleted cultures, especially in NF54-derived lines (growth rate ~4 in the absence of choline vs 7–8 in its presence) (Supplementary Fig. 4). Since reduced growth was already observed during the initial cycle of multiplication upon choline removal, it cannot be explained by increased sexual conversion, suggesting that the reduced growth reflects the metabolic consequences of the lack of choline^21^.

### The *gexp02* promoter is active in a subset of schizonts

**Figure 4:**
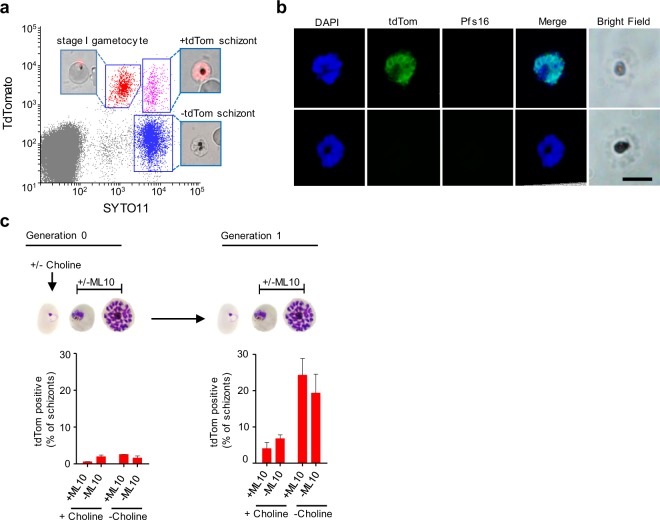
Expression of tdTom in schizonts of *gexp02-Tom* lines. (**a)** Representative flow cytometry analysis of the *NF54-gexp02-Tom* line at ~48–53 hpi (induced by choline depletion at the previous cycle and treated with ML10), showing presence of tdTom-positive schizonts (upper right gate), in addition to stage I gametocytes (upper left gate), and tdTom negative schizonts (lower right gate). Insets are live cell fluorescence microscopy images of the different subpopulations. **(b)** Representative IFA images of schizonts that are either tdTom positive or negative. All schizonts were Pfs16-negative. Scale bar: 5 µm. **(c)** Flow cytometry-based quantification of tdTom positive schizonts at the generation of induction and the following generation. Induced (choline-depleted) and uninduced (choline-supplemented) cultures, with or without addition of ML10, were tested. Data are presented as the average and s.e.m. of three independent biological replicates.

Unexpectedly, in the parasite lines *NF54-gexp02-Tom* and *E5-gexp02-Tom* we observed parasites at the schizont stage (according to their DNA content) that were positive for tdTom (Fig. 3b, top row, e.g. 48–53 hpi, and Fig. 4a). This raises the possibility that the *gexp02* promoter may be active not only in sexual rings and subsequent gametocyte stages, but also in some committed schizonts. Indeed, live cell fluorescent microscopy and IFA confirmed the occurrence of some schizonts that expressed tdTom (Fig. 4a,b). tdTom-positive schizonts were observed both in cultures with or without ML10, but they occurred infrequently in the cycle of choline removal (Generation 0) and were abundant only at the following cycle in choline-depleted cultures (Generation 1) (Fig. 4c). While some of the tdTom-positive schizonts may reflect new conversion events occurring during Generation 1, the much lower levels at Generation 0 in spite of abundant sexual commitment raise the intriguing possibility that a subset of tdTom-positive sexual rings may not develop directly into gametocytes, but rather multiply for an additional cycle as *gexp02*-expressing sexually committed forms. The higher parasitemia at Generation 1 may also influence the proportion of tdTom-positive schizonts, which unexpectedly also increased between Generations 0 and 1 in non-induced cultures. This may also reflect the inherent variability of sexual conversion rates even between consecutive generations. Of note, activation of the *gexp02* promoter doesn’t imply that the endogenous GEXP02 protein is expressed, because posttranscriptional regulation is commonly observed during sexual development^5^. Expression in a subset of schizonts of fluorescent proteins under the control of gametocyte-specific promoters has been previously observed by others^28,31,34^, and in some cases doesn’t match the expression of the endogenous protein. Future research should establish the significance of the activity of early gametocyte promoters in a subset of schizonts. However, it is important to highlight that the occurrence of tdTom-positive schizonts doesn’t affect the ability of our new transgenic lines to accurately measure sexual conversion rates, because gametocytemia can be measured long before tdTom-positive schizonts are observed (e.g. at ~20 hpi).

### *gexp02* transcripts as a marker for sexual rings

**Figure 5:**
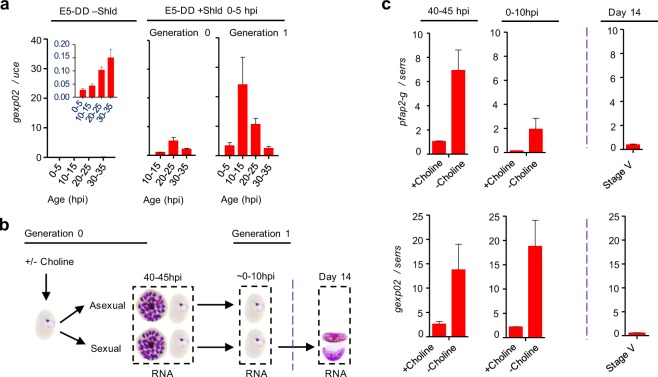
Transcriptional dynamics of the *gexp02* gene. (**a)** Transcriptional analysis of *gexp02* in the E5-HA-DD line, in which PfAP2-G is fused to the FKBP destabilization domain, cultured either without Shield 1 (Shld) (no gametocytes are formed) or with Shld added at 0–5 hpi to stabilize PfAP2-G. The inset graph in the –Shld panel shows the same data with a different y-axis range, because under these conditions *gexp02* transcripts levels were very low. Transcript levels are normalized against *ubiquitin-conjugating enzyme (uce*) (PF3D7_0812600). **(b)** Schematic overview of the design of experiments aimed at comparing the transcriptional dynamics of *pfap2-g* and *gexp02* in induced (choline-depleted) and uninduced (choline-supplemented) *NF54-gexp02-Tom* cultures. Analysis was performed at the schizont stage of the generation of induction (40–45 hpi, some new rings already present) and at the ring stage of the next generation (~0–10 hpi). Additionally, *gexp02* transcript levels were determined in mature stage V gametocytes (Day 14). **(c)** Transcript levels of *gexp02* and *pfap2-g* relative to the *serine-tRNA ligase* (*serrs*) gene (PF3D7_0717700). Data are presented as the average and s.e.m. of two independent biological replicates.

Given the very early expression observed for *gexp02*-driven reporters, we evaluated the potential use of *gexp02* transcripts as a marker for sexual rings. The ability to identify sexual rings is particularly relevant in human infections, because they are the only immature sexual stages that are present in the circulation^1,6,14,15^. To establish the specificity of *gexp02* transcripts for sexual forms, we first analyzed relative *gexp02* transcript levels in an already available collection of cDNA samples^11^ from a transgenic parasite line in which a destabilization domain is appended to PfAP2-G^3^. The protein can be stabilized by addition of the Shield 1 ligand, which results in production of gametocytes, whereas in the absence of Shield 1 no gametocytes are formed. After adding Shield 1 at 0–5 hpi, abundant *gexp02* transcripts are detected, whereas they are almost absent from untreated cultures (Fig. 5a). This result confirms that *gexp02* is expressed specifically in parasites undergoing sexual development.

Next we analyzed *gexp02* transcripts in choline-depleted and choline-supplemented cultures at a time when the majority of parasites are at the mature schizont stage but some new rings have already formed (40–45 hpi), at the ring stage of the next cycle (~0–10 hpi), and in stage V gametocytes (Fig. 5b). The latter stage was included to determine if transcripts derived from mature gametocytes can potentially have a major contribution to the total level of *gexp02* transcripts in human blood samples. In parallel, we examined the transcript levels of the sexual commitment marker *pfap2-g*. In ring stage cultures, both *pfap2-g* and *gexp02* displayed higher transcript levels in induced than in uninduced cultures, reflecting the increase in the abundance of sexual parasites. Furthermore, *gexp02* transcript levels were substantially lower in stage V gametocyte cultures than in ring stage cultures (Fig. 5c), in spite of the former being a pure sexual population whereas the latter is a mixture of sexual and asexual parasites. Based on these results, the relative contribution of mature gametocytes to the total *gexp02* transcripts in an infected human blood sample is expected to be very low. The use of different genes to normalize expression didn’t affect any of the conclusions (Supplementary Fig. 5). Altogether, these results indicate that *gexp02* transcripts provide an appropriate marker for immature sexual parasites. The higher transcript levels of *gexp02* compared to *pfap2-g* in ring stage samples suggest that *gexp02* may be a more sensitive marker for sexual rings, but a detailed analysis of *gexp02* transcripts in field samples is clearly needed before this gene can be considered a useful marker for epidemiological studies.

### Concluding remarks

The quantification of sexual stages is one of the limiting steps for an accurate determination of sexual conversion rates, which is key for studies on malaria transmission biology. Here we describe new transgenic parasite lines based on the *gexp02* promoter to quantify sexual stages. These new lines show two clear advantages over previously available tools: first, sexual parasite-specific reporter protein expression is detected as early as 10–15 hpi. This detection time enables a substantially shorter sexual conversion assay, dispensing the need to use compounds that inhibit parasite replication. Second, our genome-integrated constructs are stable and do not require drug selection pressure, which prevents potential confounding effects. Furthermore, the new transgenic lines retain the ability to respond to external conditions, which makes them suitable to study both baseline spontaneous sexual conversion, and induced conversion. Since the sequences of the *lisp1* locus targeted by the guide RNA and the homology regions in the constructs described here are highly conserved among commonly used culture-adapted parasite lines and field isolates (PlasmoDB.org), it would be straightforward to integrate the same reporter constructs in the genome of parasites of different genetic background.

We propose a sexual conversion assay that consists on culturing the *NF54-gexp02-Tom* or *E5-gexp02-Tom* lines in the presence and absence of choline (to measure basal and induced conversion, respectively), and at the next cycle measure total parasitemia and gametocytemia by flow cytometry when parasites are at the late ring or early trophozoite stage. Using this assay, the effect of different conditions (e.g. drugs) on sexual conversion rates can be assessed. The assay is robust, simple and accurate. In addition to facilitating the determination of sexual conversion rates, the new parasite lines that we have developed will enable the purification (by fluorescence-activated cell sorting) and characterization of sexual rings and early gametocytes. These transgenic lines can also be used to assess the susceptibility of early sexual forms to different drugs.

We also report that *gexp02* transcripts are highly specific for sexual stages and can be detected in sexually-developing parasites as early as the sexual ring stage. While several proteins are specifically expressed in sexual parasites, transcripts for some of these proteins appear to be more promiscuous. So far, only *pfap2-g* transcripts had been described as a mRNA marker that is specific for parasites undergoing sexual development and can be detected before stage I of gametocyte development. Further research using samples from infected humans is needed to determine the full potential of *gexp02* transcripts as a second marker for circulating immature sexual stages in epidemiological studies.

## Methods

### Parasites

The culture-adapted NF54^43,46^ and E5^3^ (a 3D7-B^47^ subclone) lines were used to generate the transgenic lines. The NF54 stock used here was obtained from Sanaria^48^. Parasites were cultured in B+ erythrocytes (3% hematocrit) in RPMI 1640-based standard medium supplemented with 10% human plasma and incubated at 37 °C under hypoxia (2% O_2_, 5.5% CO_2_, balance N_2_) and shaking (100 rpm) or static conditions. Regular culture synchronization was performed by standard sorbitol lysis, whereas for selected assays we used tight synchronization to a well-defined age window by combining Percoll purification of mature forms (followed by addition of fresh erythrocytes to establish new cultures) with sorbitol lysis 5 h later, as previously described^11^. Flow cytometry determination of parasitemia was performed essentially as described^49^.

In our laboratory, NF54 is regularly cultured in media with human serum or plasma, which contains high levels of the metabolite LysoPC that represses sexual conversion^21^, whereas E5 is regularly maintained in media with 0.5% Albumax II (with low levels of LysoPC and choline), and no human serum. Here, for most experiments we maintained both lines in Albumax II media supplemented with 2 mM choline chloride (Sigma-Aldrich no. C7527), which is analogous to supplementing with LysoPC^10,21^. To induce sexual conversion, we replaced the media by media without choline. This was always performed in combination with synchronization by sorbitol lysis, which in total involves two washing steps. Induced cultures were then maintained without choline until the end of the experiment.

For selected experiments, cultures were treated with ML10, a highly specific inhibitor of PfPKG. The compound was added at a 80 nM concentration at the trophozoite stage (typically 30–35 hpi) to inhibit merozoite egress^45^, thus preventing multiplication of asexual parasites. ML10-arrested schizonts appeared to decrease in number over time (Fig. 3b), suggesting that they progressively disintegrated.

Cultures at the ring stage (5 to 8% parasitemia) were used for transfection by electroporation with 60 µg of circular Cas9 plasmid and 30 µg of linearized donor plasmid. Starting the day after transfection, cultures were selected with 10 nM WR99210 for 4 consecutive days^50^. Diagnostic PCR analysis of genomic DNAs to validate integration of the plasmids was performed with the TaKaRa LA Taq DNA Polymerase (Takara Bio USA, Inc.). It is well established that different subclones of the same genetic background often show different phenotypes in terms of sexual conversion and ability to infect mosquitoes^3,42^. Thus, to retain the sexual conversion phenotype of the parental E5 and NF54 parasite lines, the transgenic lines obtained were not subcloned. It is likely that a subset of the parasites in the transgenic lines retained the *pDC2-Cas9-hDHFRyFCU-lisp1* plasmid and hence is resistant to WR99210. However, this plasmid contains the negative selection marker yFCU, which enables eliminating parasites containing this plasmid by selection with 5-fluorocytosine. We recommend performing this treatment before transfection of the transgenic lines with other plasmids for sequential editing, and to periodically monitor the absence of wild type parasites by PCR analysis of genomic DNA.

We defined the sexual conversion rate as the proportion of parasites that convert into sexual stages in a given parasite population^3,11^. Sexual conversion by flow cytometry or Giemsa-stained smears was calculated by dividing the gametocytemia over the initial parasitemia of a culture at the ring stage, determined when no additional replication was allowed. On the other hand, sexual conversion by IFA was measured by directly determining the proportion of Pfs16 or tdTom positive cells in >200 DAPI positive cells.

### Plasmids

Plasmid *pDC2-Cas9-hDHFRyFCU-lisp1* was based on the pDC2-Cas9-U6-hDHFRyFCU plasmid^50,51^. The single guide RNA (sgRNA) specific for the *liver-specific protein-1* gene (*lisp1*, ID: PF3D7_1418100; previous ID: PF14_0179) was generated by cloning annealed oligonucleotides (spanning positions 5,526-5,545 from the *lisp1* start codon) into a *Bbs*I site using the In-Fusion system (Clontech).

The *etramp10*.*3-tdTomato-lisp1* donor plasmid used to insert the *etramp10*.*3-tdTomato* cassette into the *lisp1* locus was based on the plasmid *164-tdTomato*^29^, which has the tdTom coding sequence under the control of the *etramp10*.*3* (ID: PF3D7_1016900; previous ID: PF10_0164) promoter and the *P*. *berghei dhfr 3*′terminator (PbDT-3′). We noticed that this plasmid contains an unexpected 162 bp fragment that includes positions 1–102 of the spliced coding sequence of a *rifin* gene (ID: PF3D7_0115300; previous ID: PFA0745W) between the *Not*I site (separates the *etramp10*.*3* promoter from tdTom) and the tdTom sequence. This fragment does not appear to interfere with the expression of the reporter gene, as correct expression was previously described^29^. This sequence is present in all plasmids generated here derived from the *164-tdTomato* plasmid. We first cloned PCR-amplified *lisp1* homology regions (HR1 and HR2) into *Kpn*I/*Sal*I and *SnaB*I/*EcoR*I sites flanking the *etramp10*.*3-tdTomato-PbDT3’* cassette. HR1 and HR2 consisted of positions 5,168-5,523 and 5,891-6,236 of the *lisp1* coding sequence (relative to the start codon), respectively.

To generate plasmid *gexp02-tdTomato-lisp1*, we replaced the *etramp10*.*3* promoter in the *etramp10*.*3-tdTomato-lisp1* plasmid with the *gexp02* promoter. The *gexp02* (ID: PF3D7_1102500; previous ID: PF11_0037) upstream region (position −2,457 to −25 bp relative to the start codon) was PCR-amplified from *P*. *falciparum* genomic DNA and cloned into plasmid *etramp10*.*3-tdTomato-lisp1* digested with *Sal*I and *Not*I using the In-Fusion system (Takara Bio USA, Inc.). An analogous procedure was followed with the *pfs16* promoter (position −863 to −1 relative to the start codon) to generate the *pfs16-tdTomato-lisp1* plasmid. All primers used to generate the plasmids are described in Supplementary Table 1.

### Immunofluorescence assay (IFA)

Blood smears for IFA were fixed with 1% paraformaldehyde in PBS for 10 min, permeabilized with 0.1% Triton X-100/PBS for 5 min, and blocked with 3% BSA in PBS for 30 min, essentially as described^11^. Two washes with PBS were performed between steps (5–10 min, shaking at 120 rpm). Primary antibodies were incubated at room temperature for 3 h or at 4 °C overnight while shaking (60 rpm). After 3 PBS washes, secondary antibodies in a solution also containing DAPI (5 µg/ml) were incubated at room temperature for 3 h while shaking (60 rpm). All antibodies were prepared in 3% BSA/PBS. The primary antibodies used were rabbit-anti-RFP (1:500; Rockland Inc. no. 600-401-379) and mouse-anti-PfS16^52^ (1:400), whereas the corresponding secondary antibodies were goat-anti-rabbit IgG-Alexa Fluor 488 (1:1000; ThermoFisher no. A11034) and donkey-anti-mouse IgG Alexa Fluor 546 (1:1000; ThermoFisher no. A10036). IFA slides were mounted with Vectashield medium (Palex Medical) and viewed under an Olympus IX51 epifluorescent microscope. Images were taken using an Olympus DP72 camera connected to CellSens Standard 1.11 software and were further processed using ImageJ software.

### Flow cytometry determination of gametocytemia

Flow cytometry was performed at several time points to capture the expression of fluorescent reporters driven by gametocyte promoters in synchronized cultures at different stages of development. Samples for flow cytometry analysis (using a BD LSRFortessa™ apparatus) were prepared by mixing the nucleic acid stain Syto11 (0.016 µM) (Life Technologies no. S7573) and 10 µl of parasite culture at 3% hematocrit in 800 µl of PBS essentially as described^49^. Flow cytometry analysis (typically 100,000 cells per sample) was set to simultaneously detect tdTom (laser: 561 nm; filter: 582/15; power: 50 mW) and Syto11 (laser: 488 nM; Filter: 525/50-505LP; power: 50 mW). Initial gating was performed using the side scatter and forward scatter areas (SSC-A versus FSC-A plot) to define the RBC population and to exclude cells or debris outside the granularity and size of RBCs. Next, singlets were gated using the forward scatter height (FSC-H) versus FSC-A plot. Sexual stages were detected on the double positive gate as parasites positive for both tdTom signal and Syto11-stained nucleic acids (Supplementary Fig. 3). Downstream analysis was performed using Flowing Software version 2.5.1 (Perttu Terho).

### Transcriptional analysis

RNA preparation and reverse-transcription quantitative PCR (RT-qPCR) were performed essentially as previously described, using a method suitable for low amounts of RNA^53^. In brief, RNA was collected in TRIzol (Invitrogen), purified with the RNeasy^®^ Mini Kit (Qiagen no. 74104), DNAse treated (Qiagen no. 79254), and cDNA synthesized using the AMV Reverse Transcription System (Promega) with a mixture of oligo (dT) and random primers. The qPCR analysis of cDNAs was performed using the standard curve method, with a standard curve for each primer pair contained in each plate. Transcript levels were normalized against transcripts of *serine-tRNA ligase* (*serrs*, ID:PF3D7_0717700), *ubiquitin-conjugating enzyme* (*uce*, ID: PF3D7_0812600), and *18S ribosomal RNA* (*18S rRNA*, IDs: PF3D7_0112300, PF3D7_1148600 and PF3D7_1371000), which show relatively stable levels across asexual and sexual blood stages (PlasmoDB.org).The primers used are described in Supplementary Table 1. Primers for *pfap2-g*, *serrs*, *uce* and *18S rRNA* are based on previously described primers^11,54^.

## Supplementary information

Supplementary Figures.

## Data Availability

Materials and protocols are available from the corresponding author on reasonable request.

## References

[1] Meibalan E, Marti M (2017). Biology of Malaria Transmission. Cold Spring Harb Perspect Med.

[2] Baker DA (2010). Malaria gametocytogenesis. Mol Biochem Parasitol.

[3] Kafsack BF (2014). A transcriptional switch underlies commitment to sexual development in malaria parasites. Nature.

[4] Poran A (2017). Single-cell RNA sequencing reveals a signature of sexual commitment in malaria parasites. Nature.

[5] Josling GA, Williamson KC, Llinas M (2018). Regulation of Sexual Commitment and Gametocytogenesis in Malaria Parasites. Annu Rev Microbiol.

[6] Nilsson SK, Childs LM, Buckee C, Marti M (2015). Targeting Human Transmission Biology for Malaria Elimination. PLoS Pathog.

[7] Brancucci NMB (2018). Probing *Plasmodium falciparum* sexual commitment at the single-cell level. Wellcome Open Res.

[8] Lopez-Rubio JJ, Mancio-Silva L, Scherf A (2009). Genome-wide analysis of heterochromatin associates clonally variant gene regulation with perinuclear repressive centers in malaria parasites. Cell Host Microbe.

[9] Cortés A, Deitsch KW (2017). Malaria Epigenetics. Cold Spring Harb Perspect Med.

[10] Filarsky M (2018). GDV1 induces sexual commitment of malaria parasites by antagonizing HP1-dependent gene silencing. Science.

[11] Bancells C (2019). Revisiting the initial steps of sexual development in the malaria parasite *Plasmodium falciparum*. Nat Microbiol.

[12] Bruce MC, Alano P, Duthie S, Carter R (1990). Commitment of the malaria parasite *Plasmodium falciparum* to sexual and asexual development. Parasitology.

[13] Kent RS (2018). Inducible developmental reprogramming redefines commitment to sexual development in the malaria parasite *Plasmodium berghei*. Nat Microbiol.

[14] Usui M (2019). *Plasmodium falciparum* sexual differentiation in malaria patients is associated with host factors and GDV1-dependent genes. Nat Commun.

[15] Pelle KG (2015). Transcriptional profiling defines dynamics of parasite tissue sequestration during malaria infection. Genome Med.

[16] Aguilar R (2014). Molecular evidence for the localization of *Plasmodium falciparum* immature gametocytes in bone marrow. Blood.

[17] Joice R (2014). *Plasmodium falciparum* transmission stages accumulate in the human bone marrow. Sci Transl Med.

[18] Carter LM (2013). Stress and sex in malaria parasites: Why does commitment vary?. Evol Med Public Health.

[19] Taylor LH, Read AF (1997). Why so few transmission stages? Reproductive restraint by malaria parasites. Parasitol Today.

[20] Bousema T, Drakeley C (2011). Epidemiology and infectivity of *Plasmodium falciparum* and *Plasmodium vivax* gametocytes in relation to malaria control and elimination. Clin Microbiol Rev.

[21] Brancucci NMB (2017). Lysophosphatidylcholine Regulates Sexual Stage Differentiation in the Human Malaria Parasite *Plasmodium falciparum*. Cell.

[22] Coleman BI (2014). A *Plasmodium falciparum* histone deacetylase regulates antigenic variation and gametocyte conversion. Cell Host Microbe.

[23] Carter R, Miller LH (1979). Evidence for environmental modulation of gametocytogenesis in *Plasmodium falciparum* in continuous culture. Bull World Health Organ.

[24] Fivelman QL (2007). Improved synchronous production of *Plasmodium falciparum* gametocytes *in vitro*. Mol Biochem Parasitol.

[25] Miao J (2013). *Plasmodium falciparum*: Generation of pure gametocyte culture by heparin treatment. Exp Parasitol.

[26] Brancucci NM (2014). Heterochromatin protein 1 secures survival and transmission of malaria parasites. Cell Host Microbe.

[27] Young JA (2005). The *Plasmodium falciparum* sexual development transcriptome: a microarray analysis using ontology-based pattern identification. Mol Biochem Parasitol.

[28] Buchholz K (2011). A high-throughput screen targeting malaria transmission stages opens new avenues for drug development. J Infect Dis.

[29] Brancucci NM, Goldowitz I, Buchholz K, Werling K, Marti M (2015). An assay to probe *Plasmodium falciparum* growth, transmission stage formation and early gametocyte development. Nat Protoc.

[30] Dixon MW, Peatey CL, Gardiner DL, Trenholme KR (2009). A green fluorescent protein-based assay for determining gametocyte production in *Plasmodium falciparum*. Mol Biochem Parasitol.

[31] Eksi S, Suri A, Williamson KC (2008). Sex- and stage-specific reporter gene expression in *Plasmodium falciparum*. Mol Biochem Parasitol.

[32] Silvestrini F (2010). Protein export marks the early phase of gametocytogenesis of the human malaria parasite *Plasmodium falciparum*. Mol Cell Proteomics.

[33] Aingaran M (2012). Host cell deformability is linked to transmission in the human malaria parasite *Plasmodium falciparum*. Cell Microbiol.

[34] Eksi S (2005). Identification of a subtelomeric gene family expressed during the asexual-sexual stage transition in *Plasmodium falciparum*. Mol Biochem Parasitol.

[35] Olivieri A, Silvestrini F, Sanchez M, Alano P (2008). A 140-bp AT-rich sequence mediates positive and negative transcriptional control of a *Plasmodium falciparum* developmentally regulated promoter. Int J Parasitol.

[36] Bruce MC, Carter RN, Nakamura K, Aikawa M, Carter R (1994). Cellular location and temporal expression of the *Plasmodium falciparum* sexual stage antigen Pfs16. Mol Biochem Parasitol.

[37] Carter R (1989). *Plasmodium falciparum*: an abundant stage-specific protein expressed during early gametocyte development. Exp Parasitol.

[38] Silvestrini F (2005). Genome-wide identification of genes upregulated at the onset of gametocytogenesis in *Plasmodium falciparum*. Mol Biochem Parasitol.

[39] Tiburcio M (2015). Specific expression and export of the *Plasmodium falciparum* Gametocyte EXported Protein-5 marks the gametocyte ring stage. Malar J.

[40] Farid R, Dixon MW, Tilley L, McCarthy JS (2017). Initiation of gametocytogenesis at very low parasite density in *Plasmodium falciparum* infection. J Infect Dis.

[41] Josling GA (2019). Regulation of sexual differentiation is linked to invasion in malaria parasites. bioRxiv (pre-review preprint).

[42] Delves MJ (2016). Routine *in vitro* culture of *P*. *falciparum* gametocytes to evaluate novel transmission-blocking interventions. Nat Protoc.

[43] Walliker D (1987). Genetic analysis of the human malaria parasite *Plasmodium falciparum*. Science.

[44] Ishino T (2009). LISP1 is important for the egress of *Plasmodium berghei* parasites from liver cells. Cell Microbiol.

[45] Baker DA (2017). A potent series targeting the malarial cGMP-dependent protein kinase clears infection and blocks transmission. Nat Commun.

[46] Ponnudurai T, Leeuwenberg AD, Meuwissen JH (1981). Chloroquine sensitivity of isolates of *Plasmodium falciparum* adapted to *in vitro* culture. Trop Geogr Med.

[47] Cortés A, Benet A, Cooke BM, Barnwell JW, Reeder JC (2004). Ability of *Plasmodium falciparum* to invade Southeast Asian ovalocytes varies between parasite lines. Blood.

[48] Roestenberg M (2013). Controlled human malaria infections by intradermal injection of cryopreserved *Plasmodium falciparum* sporozoites. Am J Trop Med Hyg.

[49] Rovira-Graells N, Aguilera-Simon S, Tinto-Font E, Cortes A (2016). New Assays to Characterise Growth-Related Phenotypes of *Plasmodium falciparum* Reveal Variation in Density-Dependent Growth Inhibition between Parasite Lines. PLoS ONE.

[50] Knuepfer E, Napiorkowska M, van Ooij C, Holder AA (2017). Generating conditional gene knockouts in *Plasmodium* - a toolkit to produce stable DiCre recombinase-expressing parasite lines using CRISPR/Cas9. Sci Rep.

[51] Lim MY (2016). UDP-galactose and acetyl-CoA transporters as *Plasmodium* multidrug resistance genes. Nat Microbiol.

[52] Moelans, I. I. M. D. Pfs16, a potential vaccine candidate against the human malaria parasite *Plasmodium falciparum*. *PhD Thesis* University of Nijmegen (1995).

[53] Mira-Martínez S (2017). Expression of the *Plasmodium falciparum* Clonally Variant *clag3* Genes in Human Infections. J Infect Dis.

[54] Rosanas-Urgell A (2010). Comparison of diagnostic methods for the detection and quantification of the four sympatric *Plasmodium* species in field samples from Papua New Guinea. Malar J.

