# Supplementary material for: Reporter lines based on the gexp02 promoter enable early quantification of sexual conversion rates in the malaria parasite Plasmodium falciparum
Source: Sci Rep. 2019 Oct 10;9:14595. doi: 10.1038/s41598-019-50768-y (PMC6787211; doi:10.1038/s41598-019-50768-y)
Supplement: Supplementary file 1 — Supplementary Figures. [file 41598_2019_50768_MOESM1_ESM.pdf]

SUPPLEMENTARY INFORMATION

Reporter lines based on the *gexp02* promoter enable early quantification of sexual conversion rates in the malaria parasite *Plasmodium falciparum*

Harvie P. Portugaliza, Oriol Llorà-Batlle, Anna Rosanas-Urgell & Alfred Cortés

Supplementary Table 1. List of oligonucleotide used in this study. Restriction sites introduced in the primers are underlined

In-Fusion® primers for cloning *lisp1* homology regions

Name	Sequence	Enzyme
lisp1_HR1_+5168_Fwd	aatactcgcggcccgGGTACC AAGACAATGGGAAATGGTGTTA	*Kpn*I
lisp1_HR1_+5523_Rev	acgttcatgcGTCGAC TTCTGGGACGACATTTATTGTT	*Sal*I
lisp1_HR2_+5891_Fwd	gcggccctgcagTACGTA TACCTATAGAGGATAAGGAGAA	*Sna*BI
lisp1_HR2_+6236_Rev	actcactataGAATTC GTAAGTGTTGTGGGTATGCTT	*Eco*RI

In-Fusion® primers for cloning *gexp02 and pfs16* promoters

Name	Sequence	Enzyme
gexp02_-2457_Fwd	cgtcccagaaGTCGAC GGATTGGCACTTATACCTTTA	*Sal*I
gexp02_-46_Rev	gctcaccatGCGGCCGC TCAAACTCTAAATGATTATCC	*Not*I
pfs16_-863_Fwd	cgtcccagaaGTCGAC CCCCTCATTTCATAGTTTGTCTT	*Sal*I
Pfs16_-1_Rev	cgtcccagaaGTCGAC GTTGAAGAAAGTATAAATAGAAAAATGGC	*Not*I

In-Fusion® primers for *lisp1* guide in *pDC2-Cas9-hDHFRyFCU-lisp1* plasmid

Name	Sequence
lisp1_guide_+5526_Fwd	taagtatataatattGAGGAACTGGGAACATGTAGgttttagagctagaa
lisp1_guide_+5545_Rev	ttctagctctaaaacCTACATGTTCCCAGTTCCTCaatattatatactta

Primers for PCR confirmation of construct integration

Name	Sequence	Remarks
lisp1_+5088_Fwd	TATGAAGAATATATTGAACGAATC	Upstream of HR1
lisp1_+6249_Rev	GATATTCATTAAACCTCTCATTG	Downstream of HR2
lisp1_+6006_Rev	AGTATACCCAGGAGTGGATAA	Within HR2
PbDT 3'_+694 _Fwd	TGTCGAAACAAAACTGGCATA	
gexp02_-46_Rev	TCAAACTCTAAATGATTATCC	

Primers for RT-qPCR

Name	Sequence	Assay
gexp02 _+813_Fwd	GGAAATTAGGAGTAAGAAGAGG	SYBR Green
gexp02_+972_Rev	TCGCTATTTATTTCACTTGGCT	
pfap2-g_+3874_Fwd	AACAACGTTCATTCAATAAATAAGG	SYBR Green
pfap2-g_+3979_Rev	ATGTTAATGTTCCCAAACAACCG	
serRS_+590_Fwd	AAGTAGCAGGTCATCGTGGTT	SYBR Green
serRS_+747_Rev	TTCGGCACATTCTTCCATAA	
uce_+67_Fwd	GGTGTTAGTGGCTCACCAATAGGA	SYBR Green
uce_+155_Rev	GTACCACCTTCCCATGGAGTA	
18srRNA_+792_Fwd	GCTTTTGAGAGGTTTTGTTACTTTG	SYBR Green
18srRNA_+1006_Rev	CTCTGACATCTGAATACGAATGC	

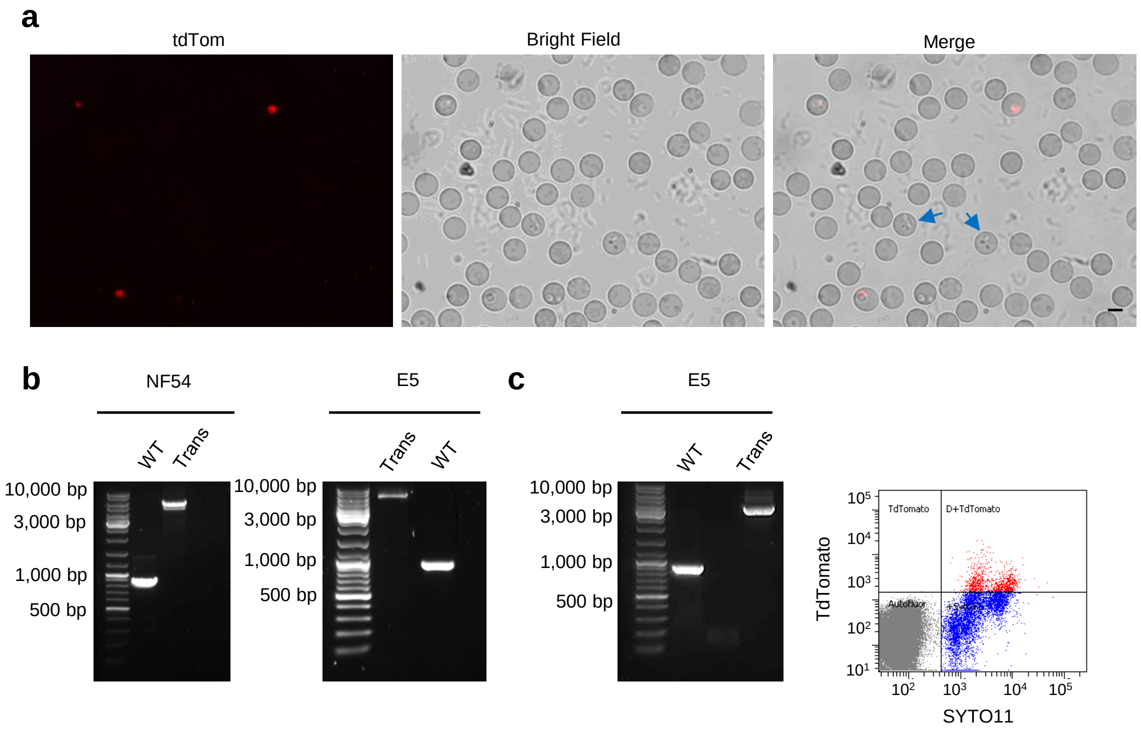

Supplementary Figure 1. Characterization of the new reporter lines. (a) Representative live cell fluorescence microscopy image of an induced (- choline) *NF54-gexp02-Tom* culture at the ring stage, as in Fig. 1c. This low magnification images show the presence of tdTom-positive rings (red fluorescence inside the RBC) and tdTom-negative rings (blue arrows). Scale bar: 5 µm. **(b)** Diagnostic PCR confirmation of integration of the *etramp10.3-tdTomato* plasmid (analogous to the *gexp02-tdTomato-lisp1* plasmid shown in Fig. 1a, but with the *etramp10.3* promoter instead of the *gexp02* promoter) at the *lisp1* locus in the NF54 and E5 lines. The forward primer from primer pair 1 (see Fig. 1a) and a reverse primer recognizing the homology region 2 (HR2) (Supplementary Table 1) were used for this PCR analysis. The amplification band in the wild-type lines (WT) is of the expected size (919 bp), and in the transgenic lines (Trans) the band is of the expected size after correct editing (5,308 bp). **(c)** Analogous analysis for integration of the *pfs16-tdTomato-lisp1* plasmid in the *lisp1* locus in the E5 line (expected size of the PCR product after correct editing: 3,964 bp), and flow cytometry-based analysis of ~24-46 h post-invasion parasites of this line showing tdTom fluorescence in late-stage asexual parasites.

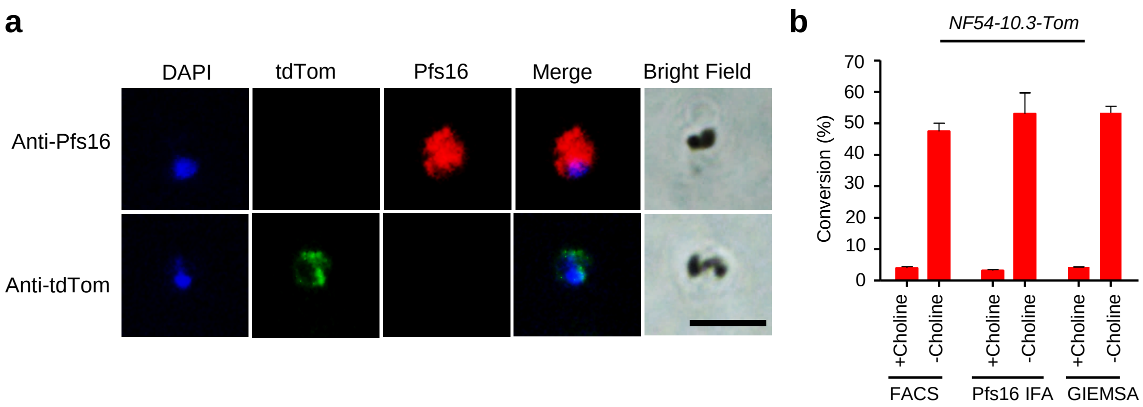

Supplementary Figure 2. Validation of the IFA assay and characterization of the *NF54-10.3-Tom* line. (a) Representative images of IFA analysis of *NF54-gexp02-Tom* stage I gametocytes to exclude cross-reactivity of secondary antibodies. Only mouse-anti-Pfs16 or only rabbit-anti-RFP primary antibodies were used in the top and bottom panels, respectively. In both cases, two secondary antibodies (goat-anti-rabbit IgG-Alexa Fluor 488 and donkey-anti-mouse IgG Alexa Fluor 546) and DAPI were used. Images are representative of 1,001 and 1,017 infected erythrocytes analyzed for anti-Pfs16 and anti-tdTom conditions, respectively. Scale bar: 5 µm. **(b)** Sexual conversion rate under +/- choline conditions of the *NF54-10.3-Tom* line as determined by flow cytometry (FACS), Pfs16 IFA, and Giemsa-stained blood smears. To determine the conversion rate by FACS, gametocytemia and initial parasitemia were measured by flow cytometry using tdTom + Syto11 and Syto11 fluorescence, respectively. Results are presented as the average and s.e.m. of two independent biological replicates.

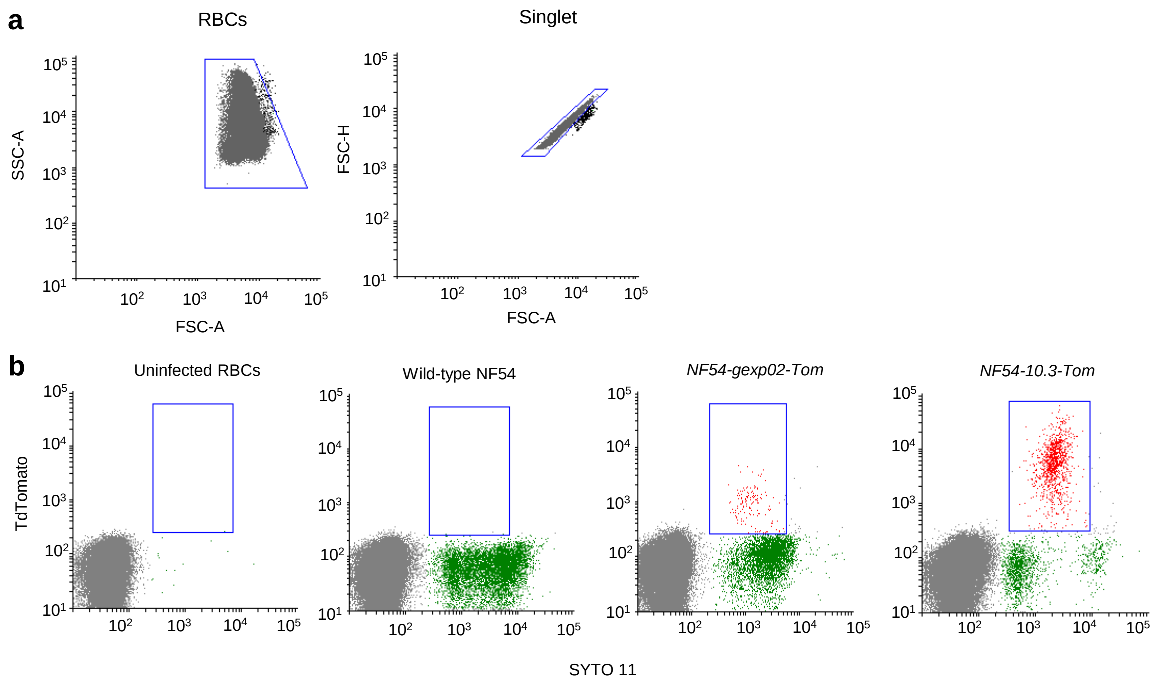

Supplementary Figure 3. Set-up of the flow cytometry assay. (a) Representative images of the initial gating strategy. The red blood cell (RBC) population was initially gated according to granularity and size (SSC-A versus FSC-A plot) and then gated to define singlets (FSC-H versus FSC-A plot). **(b)** To identify the signal attributable to parasite DNA and to tdTomato expression, we used uninfected RBCs and wild-type NF54 controls (asynchronous culture), and the transgenic lines *NF54-gexp02-Tom* (30-35hpi, + choline) and *NF54-etramp10.3-Tom* (72-94hpi, - choline) showing gametocytes in the tdTom + SYTO11 double positive gate (rectangle).

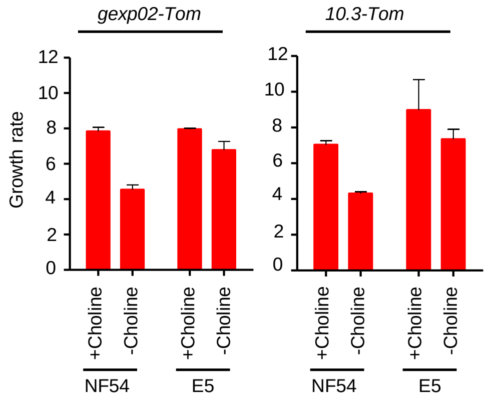

Supplementary Figure 4. Comparison of growth rates of *gexp02-Tom* and *10.3-Tom* transgenic lines under +/-choline conditions. Growth rates were calculated as the increase in parasitemia between two consecutive generations (starting with a ~1.2% parasitemia), determined by flow cytometry. Results are presented as the average and s.e.m. of two independent biological replicates.

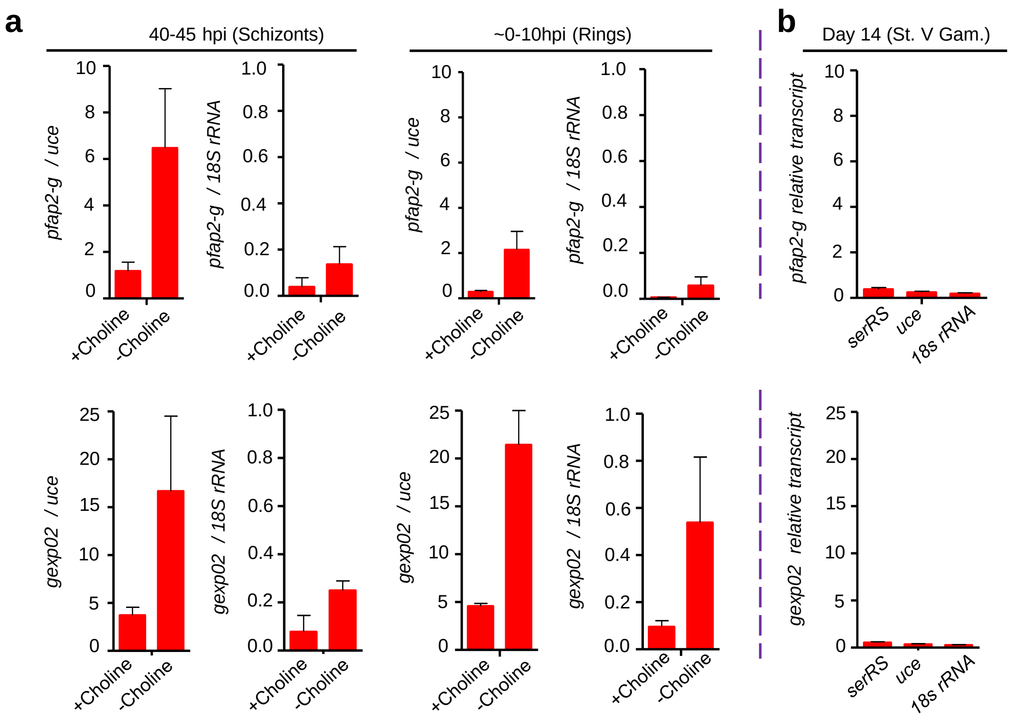

Supplementary Figure 5. Transcript levels of *gexp02* and *pfap2-g* normalized against different reference genes. (a) Relative transcript levels of *pfap2-g* and *gexp02* in induced (- choline) and uninduced (+ choline) *NF54-gexp02-Tom* cultures analyzed at the schizont (40-45 hpi) and next generation ring (~0-10 hpi) stages. Samples are the same as in Fig. 5c in the main text, but normalized against transcript levels of *ubiquitin-conjugating enzyme* (*uce*, ID: PF3D7_0812600), *serine-tRNA ligase* (*serrs*, ID:PF3D7_0717700) or *18S ribosomal RNA* (*18S rRNA*, IDs: PF3D7_0112300, PF3D7_1148600 and PF3D7_1371000). **(b)** Transcript levels of *pfap2-g* and *gexp02* in stage V gametocytes, as in Fig. 5c in the main text, but normalized against the different genes indicated. Results are presented as the average and s.e.m. of two independent biological replicates.

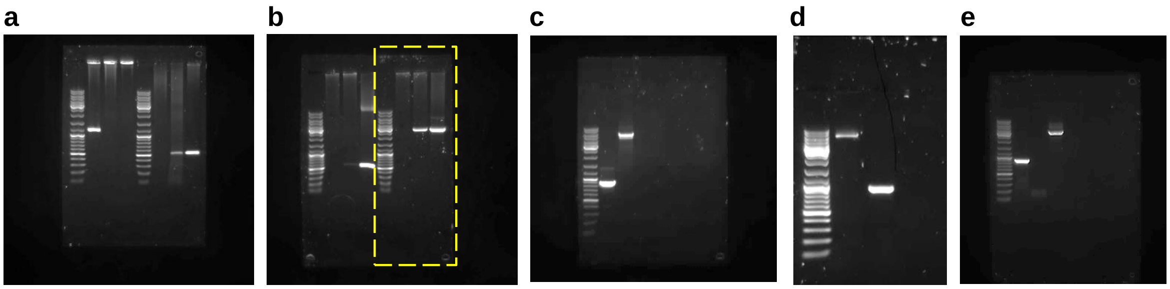

Supplementary Figure 6. Full length gels. (a) Full length gel for Fig. 1b (PCRs 1 and 3). **(b)** Full length gel for Fig. 1b (PCR 2). The dashed line indicates the part of the gel containing the relevant samples. **(c)** Full length gel for Supplementary Fig. 1a (left). **(d)** Full length gel for Supplementary Fig. 1a (right). **(e)** Full length gel for Supplementary Figure 1b.
